# Diagnostic Performance of MRI for Posterolateral Meniscocapsular Detachment in ACL-Injured Knees: A Retrospective Study

**DOI:** 10.3390/diagnostics16152343

**Published:** 2026-07-27

**Authors:** Tommaso Bonanzinga, Elena Tosi, Alberto Favaro, Emanuela Morenghi, Nicola Magarelli

**Affiliations:** 1 Department of Biomedical Sciences, Humanitas University, Via Rita Levi Montalcini 4, 20072 Milan, Italy; tommaso.bonanzinga@hunimed.eu (T.B.); emanuela.morenghi@humanitas.it (E.M.); 2 IRCCS Humanitas Research Hospital, Via Manzoni 56, 20089 Milan, Italy; alberto.favaro@humanitas.it; 3 Department of Diagnostic Imaging, IRCCS Humanitas Research Hospital, Via Manzoni 56, 20089 Milan, Italy; nicola.magarelli@humanitas.it

**Keywords:** posterolateral corner, meniscocapsular detachment, MRI, ACL, popliteomeniscal fascicles, knee, diagnostic accuracy, arthroscopy

## Abstract

**Background/Objectives:** Posterolateral meniscocapsular detachment represents a potentially underrecognized component of knee trauma in patients with anterior cruciate ligament (ACL) injury. Altered rotational kinematics in ACL-deficient knees may increase stress on posterolateral corner structures. Although an association between ACL tears and posterolateral abnormalities has been reported, the true diagnostic performance of magnetic resonance imaging (MRI) for these lesions remains incompletely defined. This study aimed to evaluate the diagnostic accuracy of MRI for posterolateral meniscocapsular detachment in patients with confirmed ACL injury, to describe associated meniscal tears, and to assess the association between lateral tibial plateau bone marrow edema, age, and sex with the presence of detachment. **Methods:** In this retrospective study, 117 knee MRI examinations (1.5 T and 3 T) of patients with native ACL rupture or ACL graft failure were reviewed. Two readers independently assessed posterolateral meniscocapsular detachment. Ramp lesions, other meniscal tears and lateral compartment bone marrow edema were evaluated in consensus. Arthroscopy served as the reference standard. Diagnostic performance and inter-observer agreement were calculated. Detachment was considered present only when both readers independently identified the lesion. **Results:** MRI demonstrated high specificity (90.8%; 95% CI: 83.7–95.5%) for posterolateral meniscocapsular detachment, and promising sensitivity (87.5%; 95% CI: 47.3–99.7%) based on a small number of confirmed cases (*n* = 8); this estimate should be interpreted with caution given the wide confidence interval. One arthroscopically confirmed lesion was missed, and false-positive findings were limited. MRI also showed good diagnostic performance for ramp lesions and other associated meniscal tears, assessed as secondary endpoints. Lateral tibial plateau bone marrow edema, age, and sex were not significantly associated with detachment. **Conclusions:** Routine MRI shows high specificity and promising, although preliminary, sensitivity for detecting posterolateral meniscocapsular detachment in ACL-injured knees; given the small number of confirmed cases, this estimate warrants confirmation in larger cohorts. Careful multiplanar assessment and detailed anatomical knowledge are essential to optimize detection. Accurate preoperative MRI characterization may help guide surgical planning and reduce the risk of missed posterolateral meniscocapsular pathology at arthroscopy.

## 1. Introduction

Anterior cruciate ligament (ACL) tears account for a considerable proportion of sports-related knee injuries requiring surgical management, and their sequelae extend well beyond the ligament itself. Concomitant capsuloligamentous and meniscal injuries are common, and failure to recognise them preoperatively may compromise rotational stability and long-term outcomes [1,2]. In this context, growing interest has focused on the posterolateral corner (PLC) as a critical secondary stabiliser that resists tibial external rotation and varus stress, functions that become mechanically relevant precisely when the ACL is deficient [1,3].

**Figure 1:**
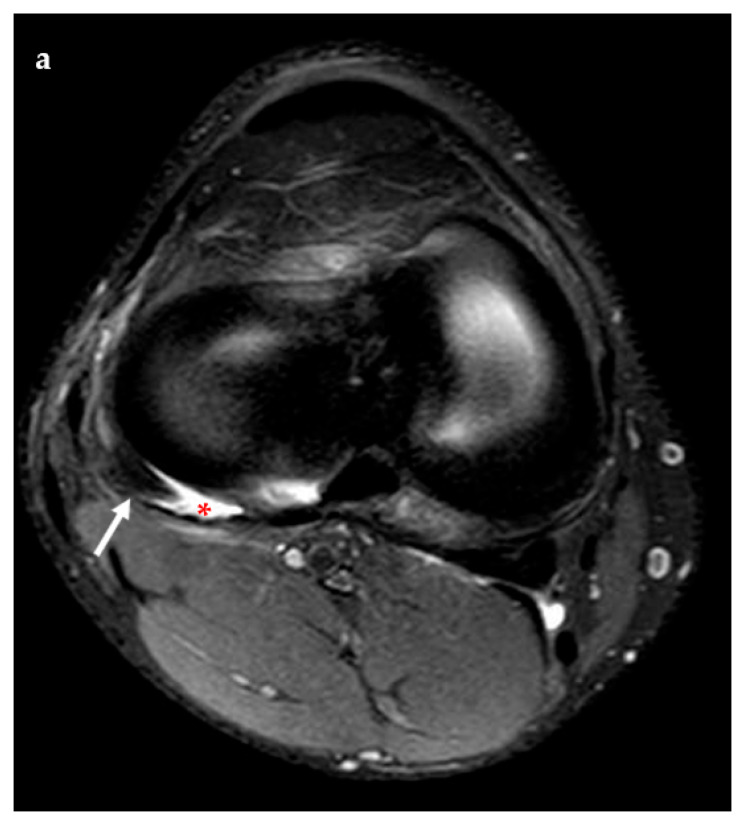

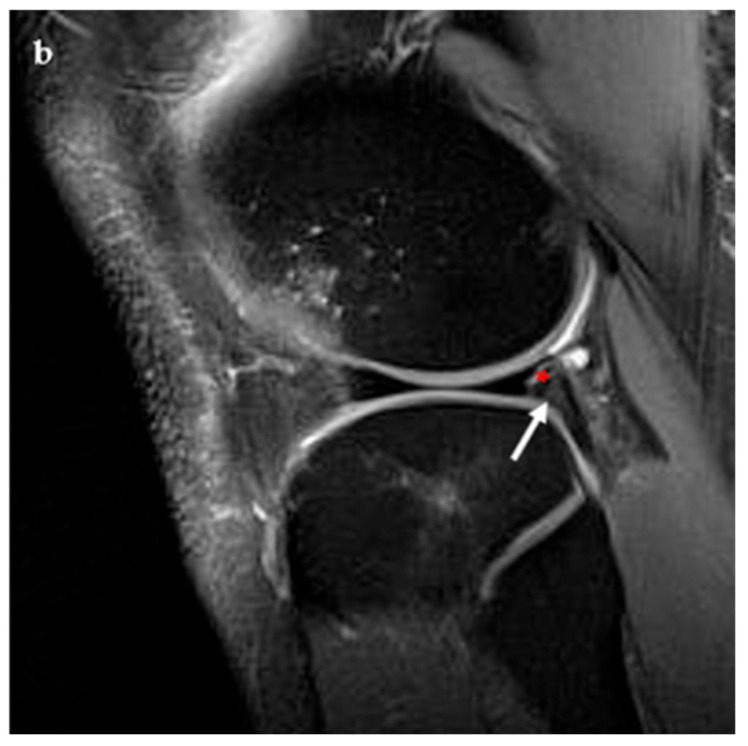
(**a**) Axial proton density-weighted 1.5-T MRI image and (**b**) sagittal proton density-weighted 3-T MRI image demonstrating the popliteus hiatus (red asterisk) and the popliteus tendon (arrow) coursing through it.

The PLC comprises a complex and variably described anatomical network, including the popliteus tendon, the popliteofibular ligament, the arcuate ligament, the fibular collateral ligament, and the popliteomeniscal fascicles (PMFs). Among these structures, the PMFs play a specific role in stabilizing the lateral meniscus within the popliteal hiatus (Figure 1), ensuring controlled meniscal movement during knee flexion and extension [2,4,5]. The popliteal hiatus is a physiological gap in the posterior capsule of the knee through which the popliteus tendon passes, and the PMFs form its superior and inferior boundaries, anchoring the posterior horn of the lateral meniscus to the musculotendinous region of the popliteus.

**Figure 2:**
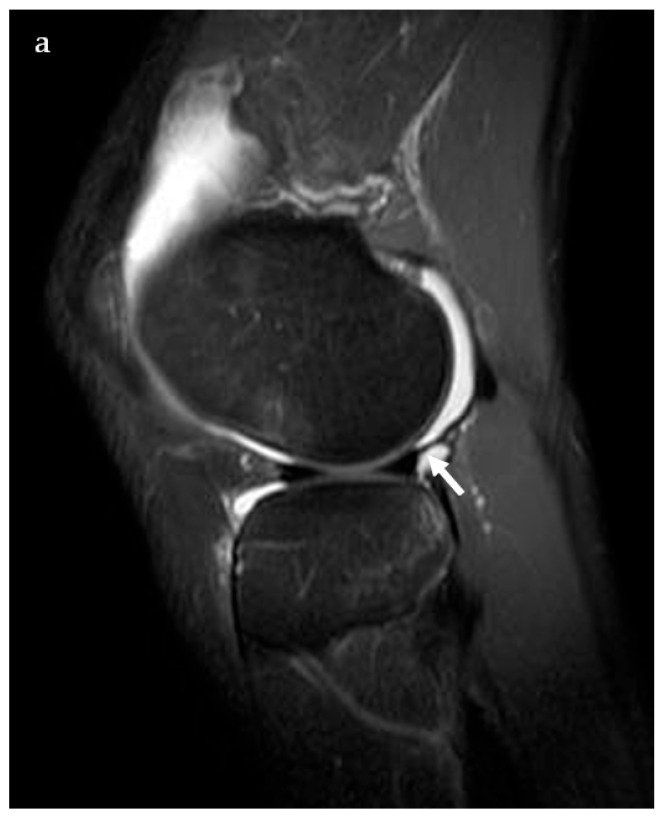

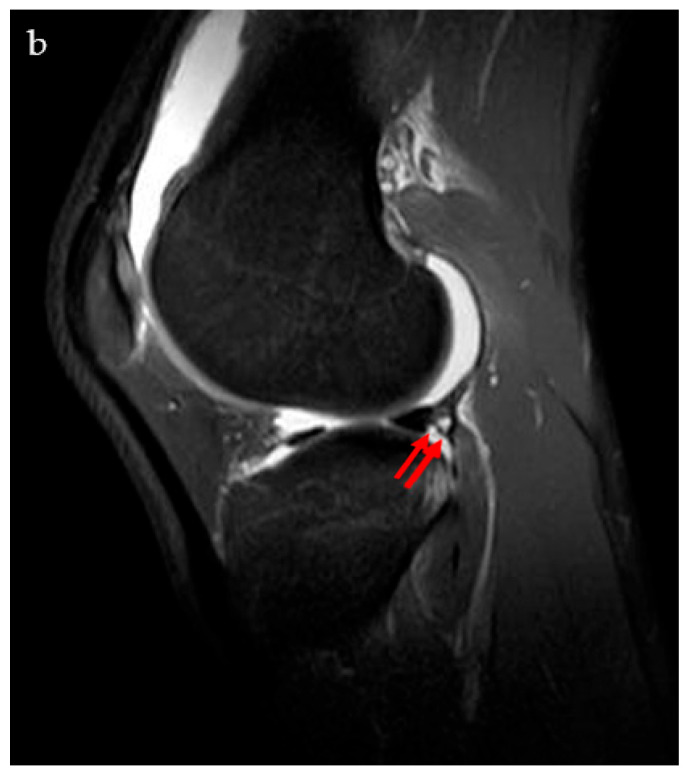
(**a**) Sagittal proton density-weighted (PDw) magnetic resonance (1.5 T) demonstrating the posterosuperior meniscopopliteal fascicle (psPMF) (white arrow), seen as a thin low-signal-intensity band extending from the posterior horn of the lateral meniscus to the popliteus tendon within the popliteomeniscal fascicular complex. (**b**) Sagittal proton density-weighted (PDw) magnetic resonance image acquired at 1.5 T showing the posteroinferior meniscopopliteal fascicle (piPMF) (red arrow), appearing as a fine hypointense band arising from the inferior edge of the posterior horn of the lateral meniscus and running obliquely toward the popliteus musculotendinous structures at the lower margin of the popliteal hiatus.

The popliteomeniscal fascicles are classically described as comprising three distinct components: the anteroinferior (aiPMF), the posterosuperior (psPMF), and the posteroinferior (piPMF) fascicles (Figure 2) [4,6,7]. The three components differ considerably in consistency and detectability: the psPMF is the most reliably identified on MRI, the aiPMF is anatomically variable and may be absent altogether, and the piPMF—identified in only 40–60% of cadaveric specimens—follows an oblique course that predisposes it to partial volume averaging and a pseudotear appearance [4,6,7].

**Figure 3:**
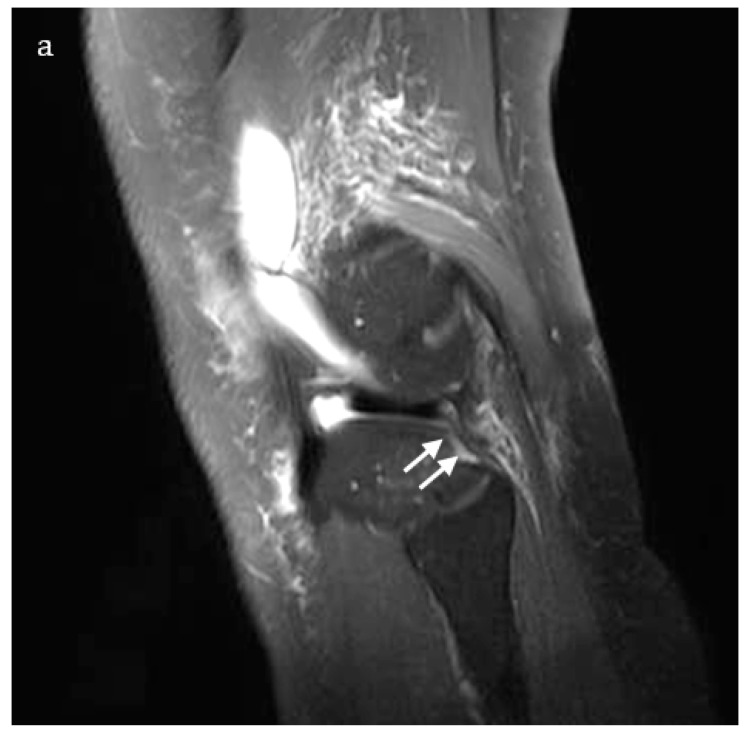

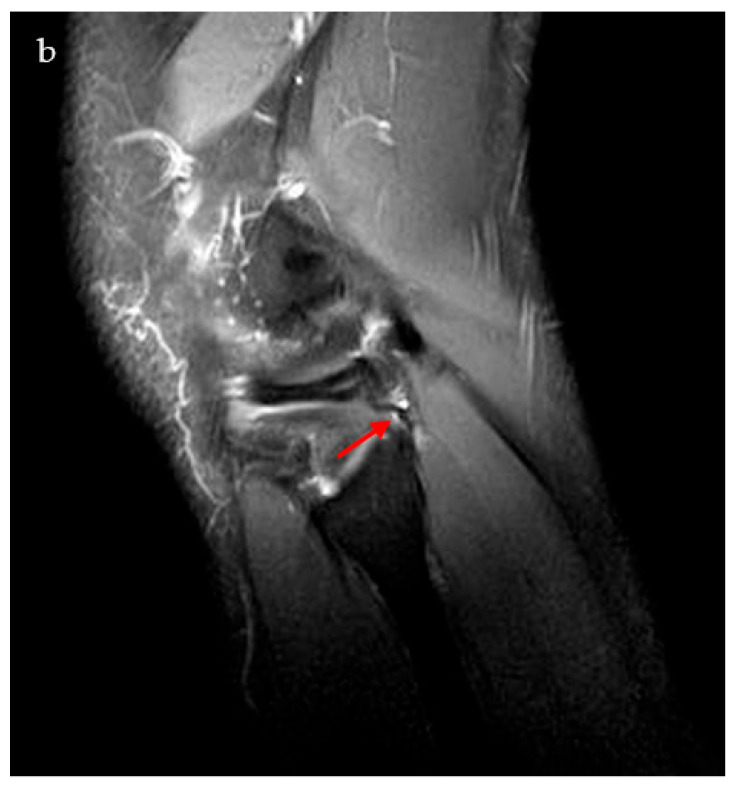
(**a**) Sagittal proton density-weighted (PDw) magnetic resonance image (1.5 T) demonstrating the anteroinferior meniscopopliteal fascicle (aiPMF) (white arrows), which courses obliquely from the posterior horn of the lateral meniscus toward the popliteus tendon, contributing to stabilization of the popliteomeniscal complex. The aiPMF is often thin and may not be consistently identifiable on routine MRI examinations. (**b**) Sagittal PDw MRI showing rupture of the anteroinferior meniscopopliteal fascicle, which appears elongated and lax (red arrow), associated with a concomitant tear of the posterior horn of the lateral meniscus.

Functionally, the PMFs are the principal restraints of the lateral meniscus posterior horn at 90° of flexion, resisting its pathological anterior translation under load [5]. When these fascicles are disrupted—whether in isolation (Figure 3) or as part of a more extensive ACL or PLC injury—the lateral meniscus becomes hypermobile, and patients may report lateral pain, a catching or locking sensation, and difficulty with squatting. If the instability goes unrecognised, recurrent abnormal meniscal translation can trigger secondary tears and progressive chondral damage, ultimately accelerating lateral compartment osteoarthritis [8,9].

Several studies have demonstrated that PLC abnormalities, including tears of the PMFs, are not uncommon in ACL-injured knees, with some series reporting PMF lesions in up to 80% of patients with acute ACL and/or PLC injury [8,10,11,12]. However, reported incidence rates vary widely depending on imaging criteria and surgical confirmation. Most investigations have focused on major PLC components such as the fibular collateral ligament and popliteofibular ligament, while the true prevalence and diagnostic accuracy of MRI for detecting subtle posterolateral meniscocapsular detachment remain incompletely characterized [13].

MRI represents the established non-invasive reference modality for assessing ACL injuries and associated meniscal pathology. While its diagnostic performance for major meniscal tears is well established, its reliability in detecting subtle meniscocapsular or popliteomeniscal lesions remains uncertain, particularly in the absence of standardized imaging criteria and in the presence of inter-reader variability [5,13]. Some authors suggest that unrecognized posterolateral lesions may contribute to persistent instability or graft overload following ACL reconstruction [3,13]. Furthermore, lateral compartment bone marrow edema—commonly associated with pivot-shift injury mechanisms—has been proposed as a potential indirect marker of concomitant posterolateral soft-tissue injury, although this relationship remains debated [14].

We hypothesised that, despite the absence of dedicated posterolateral-oriented sequences in routine clinical protocols, MRI would show good diagnostic accuracy for posterolateral meniscocapsular detachment when interpreted with awareness of its subtle imaging features, and that inter-observer agreement for this finding would be high even between readers with different levels of experience. The primary objective of this study was therefore to evaluate the diagnostic accuracy of 1.5 T and 3 T MRI in detecting posterolateral meniscocapsular detachment in patients with confirmed ACL injury, using arthroscopy as the reference standard, and to quantify inter-observer agreement for this finding. Because the same MRI examinations and arthroscopic reports already provided this information, we additionally recorded ramp lesions, other meniscal tears, and lateral compartment bone marrow edema as secondary endpoints, rather than as a separate focus of the study.

## 2. Materials and Methods

### 2.1. Study Design and Population

This retrospective observational study was conducted at Istituto Clinico Humanitas (Humanitas Research Hospital). The institutional MRI database and electronic medical records were systematically screened to identify eligible patients.

Inclusion criteria were: (i) age 10–60 years; (ii) confirmed ACL rupture or ACL graft failure; and (iii) availability of a diagnostic-quality knee MRI performed at 1.5 T or 3 T at our institution. Patients with ACL graft failure were included alongside those with native ACL rupture because the posterolateral meniscocapsular complex is anatomically distinct from the intra-articular ACL graft and its fixation hardware, which is not expected to interfere with assessment of the popliteal hiatus region; this assumption is discussed further as a limitation.

Exclusion criteria included: (i) incomplete MRI acquisition or inadequate image quality (<1.5 T); (ii) prior surgery significantly altering native knee morphology (excluding ACL reconstruction when applicable); and (iii) previous treatment potentially modifying joint anatomy in a manner interfering with imaging evaluation.

After application of selection criteria, 117 MRI examinations were included in the final analysis. All data were anonymized prior to evaluation. Written consent for anonymized data use for scientific purposes was obtained from all patients. The study was conducted in accordance with the Declaration of Helsinki and approved by the local Ethics Committee of Humanitas Research Hospital (No. 23/26; 16 June 2026).

### 2.2. MRI Protocol

MRI examinations were performed using 1.5 T and 3 T scanners with standardized knee protocols. Of the 117 examinations included in the diagnostic accuracy analysis, 97 (82.9%) were acquired at 1.5 T and 20 (17.1%) at 3 T. MRI protocols included T1-weighted, T2-weighted, proton density-weighted (PD), and short tau inversion recovery (STIR) sequences acquired in the sagittal, coronal, and axial planes. Non-fat-suppressed sequences were also obtained for anatomical delineation. In some cases, the coronal plane was acquired using a T2-weighted gradient-echo sequence. Slice thickness ranged from 3 to 4 mm across standard orthogonal planes. No dedicated posterolateral corner sequences or targeted thin-slice acquisitions of the posterolateral compartment were acquired at either field strength, reflecting routine clinical practice; a summary of the imaging protocol by field strength is provided in Table 1. Given the limited number of confirmed lesions (*n* = 8) and their uneven distribution between the two field strengths, a formal subgroup comparison of diagnostic performance by magnetic field strength was not statistically meaningful and was not performed; this is noted as a limitation below.

### 2.3. Image Analysis

Two radiologists with markedly different levels of musculoskeletal experience independently reviewed all MRI examinations: a senior musculoskeletal radiologist with approximately 30 years of experience, and a radiology resident in the final year of training. Both were blinded to arthroscopic findings. Independent evaluation was limited to the primary endpoint: presence or absence of posterolateral meniscocapsular detachment. Inter-observer agreement was calculated. This deliberate pairing of a highly experienced reader with a trainee was intended to test whether reproducible identification of this subtle finding is achievable across a realistic range of clinical experience, rather than only among expert observers.

In cases of disagreement, no forced consensus was performed. For diagnostic performance analysis, posterolateral meniscocapsular detachment was considered present only when both readers independently identified the lesion on MRI. Ramp lesions were assessed in consensus and recorded as present or absent. The presence or absence of bone marrow edema at the lateral tibial plateau was also evaluated in consensus. Other meniscal tears (medial and lateral) were evaluated descriptively and categorized according to their location and pattern. The concordant-reader criterion for the primary endpoint was chosen a priori as a conservative reference definition, intended to minimise the risk of false-positive detachment given the subtlety of this finding and the absence of a forced-consensus step; we considered this preferable to attributing the diagnosis to a single reader’s call in cases of disagreement. We recognise that this choice is conservative by construction and is expected to reduce apparent sensitivity while increasing apparent specificity, and we report this explicitly as a methodological limitation. Reader-specific diagnostic performance was not analysed as a separate endpoint in the original study design; of the 5 discordant cases, disagreement most often involved one reader identifying a subtle finding that the other did not, rather than diametrically opposite calls.

### 2.4. MRI Diagnostic Criteria for Posterolateral Meniscocapsular Detachment

No universally standardized MRI criteria exist for this diagnosis. Readers were guided by the imaging features summarized in Table 2, derived from the anatomical and imaging literature on the popliteomeniscal fascicular complex [4,6]. Particular attention was paid to distinguishing true detachment from the pseudotear appearance that can result from partial volume averaging of the obliquely oriented posteroinferior fascicle, by requiring corroboration across multiple imaging planes rather than reliance on a single sagittal or coronal image.

### 2.5. Arthroscopic Correlation

Arthroscopy served as the reference standard for the diagnosis of posterolateral meniscocapsular detachment and ramp lesions. Surgical reports were retrospectively reviewed and correlated with MRI findings. Only patients with documented arthroscopic evaluation of the posterolateral compartment were included in the diagnostic performance analysis.

All arthroscopic procedures were performed using standard anteromedial and anterolateral portals according to routine clinical practice. During surgery, the lateral meniscus was routinely inspected, and its stability was systematically assessed by probing the posterior horn and evaluating meniscal laxity.

Posterolateral meniscocapsular detachment was considered present when the operative report documented abnormal meniscocapsular separation associated with pathological mobility of the lateral meniscus during probing. Arthroscopic procedures were performed by different orthopedic surgeons. Surgeons were not blinded to the preoperative MRI findings, as review of preoperative imaging is an integral part of routine surgical planning and withholding this information would not have been ethically appropriate. Arthroscopic findings documented in the operative reports were used as the reference standard for comparison with MRI findings.

### 2.6. Statistical Analysis

Diagnostic performance metrics—including sensitivity and specificity, positive predictive value (PPV), negative predictive value (NPV), diagnostic accuracy, positive and negative likelihood ratios (LR+ and LR−)—were calculated using arthroscopy as the reference standard, along with their 95% confidence intervals (95% CIs). Inter-observer agreement for the primary endpoint was assessed using Cohen’s kappa coefficient. Associations between posterolateral meniscocapsular detachment and clinical or imaging variables (sex, age, and lateral tibial plateau bone marrow edema) were evaluated using appropriate statistical tests for comparison between groups. A *p*-value < 0.05 was considered statistically significant.

## 3. Results

### 3.1. Study Population

A total of 121 MRI examinations were initially reviewed. Four cases were excluded from diagnostic performance analysis due to the absence of corresponding arthroscopic data (2 cases) and because ACL rupture was not confirmed at surgery (2 cases). Therefore, 117 patients were included in the final diagnostic accuracy analysis. The study population consisted of 77 males and 44 females, with a mean age of 30.5 years (range: 13–57 years).

### 3.2. Arthroscopic Findings

At arthroscopy, posterolateral meniscocapsular detachment was identified in 8 of 117 patients (6.8%), while 109 patients (93.2%) showed no evidence of such lesions. Ramp lesions were identified in 28 patients (23.9%), whereas 89 patients (76.1%) had no ramp lesion at surgery. Concomitant meniscal tears were described in 76 patients (65%), involving the medial meniscus in 44 cases, the lateral meniscus in 21 cases and both menisci in 11 cases.

### 3.3. Inter-Observer Agreement for Posterolateral Meniscocapsular Detachment

Independent assessment of posterolateral meniscocapsular detachment resulted in agreement in 112 of 117 cases (95.7%), with disagreement observed in 5 cases (4.3%). Inter-observer agreement was excellent, with a Cohen’s kappa coefficient of 0.846, indicating near-perfect reproducibility between readers. This high level of agreement is particularly relevant given the subtle anatomy and small size of the posterolateral meniscocapsular attachments. Despite the intrinsic difficulty in detecting these lesions on routine MRI, interpretation proved highly consistent across observers with different levels of experience.

### 3.4. Diagnostic Performance of MRI for Posterolateral Meniscocapsular Detachment

Using arthroscopy as the reference standard and a concordant-reader model (MRI positive only when both readers agreed), MRI demonstrated sensitivity of 87.5% (95% CI: 47.3–99.7%) and specificity of 90.8% (95% CI: 83.7–95.5%). Seven of 8 arthroscopically confirmed lesions were correctly identified (true positives), with one false-negative case. Among patients without surgical evidence of detachment, 99 of 109 were correctly classified (true negatives), while 10 cases were false positives. This leads to a PPV of 41.2% (95% CI 18.4–67.1%), an NPV of 99.0% (95% CI 94.6–100%), an LR+ of 9.56 (95% CI 5.00–18.20) and an LR− of 0.14 (95% CI 0.02–0.86), with an accuracy of 90.6%. Given the low prevalence (6.8%), confidence intervals for sensitivity and PPV were relatively wide. The full contingency table and diagnostic indices are summarized in Table 3.

### 3.5. Diagnostic Performance of MRI for Ramp Lesions

For ramp lesions, MRI demonstrated: sensitivity of 78.6% (22/28) and specificity of 87.6% (78/89). MRI correctly identified 22 of 28 arthroscopically confirmed ramp lesions, with 6 false-negative cases. Eleven false-positive findings were observed among patients without surgical confirmation. This leads to a PPV of 66.7% (95% CI 48.2–82.0%), an NPV of 92.9% (95% CI 85.1–97.3%), an LR+ of 6.36 (95% CI 3.54–11.42), and an LR− of 0.24 (95% CI 0.12–0.50). These results are consistent with the current literature reporting moderate sensitivity and good-to-excellent specificity for ramp lesion detection on routine MRI [15]. The contingency table for ramp lesion assessment is provided in Table 4.

### 3.6. Concomitant Meniscal Tears

Assessment of additional meniscal tears (medial and lateral) showed complete diagnostic concordance with arthroscopy in 80 of 117 cases (68.4%), partial concordance in 16 cases (13.7%) and discordant findings in 21 cases (17.9%). The distribution of concordance outcomes is detailed in Table 5.

### 3.7. Lateral Compartment Bone Marrow Edema

Bone marrow edema of the lateral compartment was observed in 81 of 117 patients (69.2%). Among patients with arthroscopically confirmed posterolateral meniscocapsular detachment, 6 of 8 patients (75.0%) demonstrated lateral tibial plateau edema, compared with 75 of 109 patients (68.8%) without such lesions. This difference was not statistically significant (*p* = 1.000) (Table 6).

### 3.8. Association with Sex and Age

Among patients with posterolateral meniscocapsular detachment, 6 of 8 (75%) were male, compared with 73 of 109 (67.0%) in the non-lesion group. No statistically significant association between sex and presence of posterolateral detachment was observed (*p* = 1.000). The mean age was 30.7 years in patients with posterolateral detachment and 28.8 years in those without, with no statistically significant difference (*p* = 0.433). The associations between clinical variables and detachment are summarized in Table 6.

## 4. Discussion

In this retrospective series of 117 ACL-injured knees, routine MRI at 1.5 T and 3 T achieved good sensitivity and high specificity for posterolateral meniscocapsular detachment when a strict concordant-reader criterion was applied. Inter-observer agreement was excellent (kappa 0.846), which is not a trivial finding given the small dimensions and subtle imaging features of the PMF complex: it suggests that, with appropriate awareness, consistent lesion identification is achievable even across readers with different levels of musculoskeletal experience.

The diagnostic accuracy data should be interpreted in the context of lesion prevalence: with only 8 confirmed detachments in 117 cases (6.8%), the 95% CI for sensitivity spans a wide range, and the point estimate of 87.5% rests on a single missed case. That said, the specificity of 90.8% was estimated on a much larger denominator and can be considered robust. The false-negative case is a reminder that even experienced readers may overlook marginal lesions, particularly when standard-thickness sequences are the only available tool.

For ramp lesions, which were evaluated as a secondary endpoint, MRI demonstrated moderate sensitivity (78.6%) and good specificity (87.6%), in line with pooled estimates from recent systematic reviews [15,16]. This region is also inherently challenging to assess, owing to its posterior location and the thin fibers at the meniscocapsular junction [15,17]; the imperfect sensitivity observed reinforces the recommendation that the posteromedial compartment be systematically explored at arthroscopy regardless of preoperative MRI findings [15].

Lateral tibial plateau bone marrow edema, present in 69% of the cohort, is consistent with the predominance of high-energy pivot-shift mechanisms in ACL-injured knees [14]. We had hypothesised that this finding might correlate with posterolateral capsular injury, but no statistically significant association was found, mirroring other series [14]. Likewise, neither age nor sex was associated with detachment. Taken together, none of these readily available clinical or imaging variables proved useful as a proxy for PMF disruption, underscoring that direct MRI assessment of the posterolateral compartment itself remains necessary.

Posterolateral meniscocapsular lesions should not be regarded as isolated imaging curiosities but rather as part of the rotational injury spectrum associated with ACL rupture. From a biomechanical standpoint, these lesions are thought to occur predominantly during pivot-shift mechanisms involving valgus stress combined with internal tibial rotation and anterior tibial translation. Such forces generate excessive stress within the posterolateral stabilizing structures of the knee, including the popliteomeniscal fascicles and the adjacent capsular attachments. In clinical practice, suspicion for posterolateral meniscocapsular injury should therefore be increased in patients presenting with high-grade pivot-shift phenomena, persistent lateral joint-line pain, painful squatting, or mechanical symptoms such as catching and locking despite otherwise apparently isolated ACL injury. These symptoms may persist even after technically successful ACL reconstruction if concomitant posterolateral instability remains untreated.

Moreover, subtle posterolateral instability may be difficult to appreciate during standard clinical examination, particularly in the acute post-traumatic setting where pain and joint effusion limit dynamic assessment. As a result, MRI frequently represents the only preoperative opportunity to identify these lesions. For this reason, radiologists should maintain a high index of suspicion in ACL-deficient knees demonstrating lateral meniscal abnormalities adjacent to the popliteal hiatus, especially when associated with peripheral posterior horn signal alteration or unexplained irregularity of the popliteomeniscal fascicular complex.

As outlined in the Introduction, the central diagnostic challenge is the anatomical variability of the three PMFs—particularly the oblique, poorly aligned course of the posteroinferior fascicle, which predisposes it to partial volume averaging and an artefactual appearance of discontinuity even in intact knees [4,6].

This anatomical complexity is compounded by routine protocol constraints: the 3–4 mm slice thickness used in this series, while adequate for the cruciate ligaments and meniscal body, is genuinely insufficient for structures only a few millimetres across. Dedicated thin-slice oblique sequences have been shown to improve fascicle delineation [4] but were not used here, and their routine adoption would likely reduce both false-positive and false-negative rates in PMF assessment.

The potential clinical consequences of an unrecognised posterolateral meniscocapsular lesion are worth considering, although we did not evaluate postoperative outcomes in this cohort and the following should be regarded as hypotheses derived from the broader literature rather than as findings of this study. When PMF detachment is not identified before surgery, ACL reconstruction proceeds without stabilisation of the lateral meniscus, and prior reports suggest this may be associated with persistent lateral knee pain, catching or locking, and painful squatting postoperatively [8,9,18,19]. It has also been proposed that resulting meniscal hypermobility could increase susceptibility to secondary meniscal tears and lateral compartment cartilage damage over time [8,20], and that unaddressed posterolateral instability could theoretically transmit additional load to the ACL graft [3,13,21,22]. These associations remain unconfirmed in our data and would require dedicated prospective studies with postoperative follow-up to establish.

For context, PLC injuries in general are reportedly missed at initial presentation in up to 72% of cases [23,24]; the PMF detachments in our series are subtler still, but the underlying principle is the same—what is not recognised preoperatively cannot be addressed at surgery.

**Figure 4:**
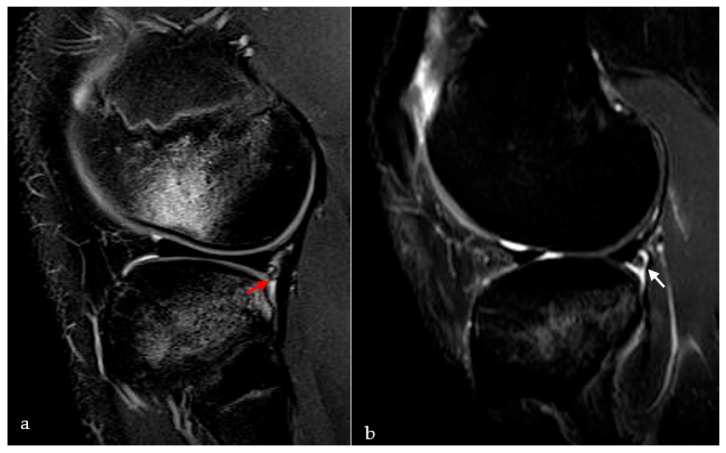
(**a**) Sagittal 3 T post-traumatic knee MRI demonstrating a diagnostic pitfall related to the posteroinferior meniscopopliteal fascicle (piPMF), which appears tortuous and elongated (red arrow), potentially mimicking structural abnormality despite preservation of fascicular continuity. (**b**) Sagittal 1.5 T knee MRI showing another diagnostic pitfall in which the piPMF appears focally interrupted (white arrow), simulating a fascicular tear; this finding is related to the complex oblique course and thin anatomy of the structure rather than to true injury.

One pitfall that we encountered repeatedly in this cohort is the pseudotear appearance of the posteroinferior popliteomeniscal fascicle (Figure 4). Owing to its oblique course and thin morphology, this structure may appear discontinuous on routine coronal or sagittal sequences, mimicking a tear. This pseudodisruption is frequently attributable to partial volume averaging rather than true structural injury, and systematic multiplanar assessment is therefore essential to avoid overdiagnosis and unnecessary surgical intervention [4]. The relatively high number of false positives observed in our cohort (10 cases) is consistent with this phenomenon and highlights the need for awareness of this pitfall among radiologists evaluating ACL-injured knees.

This study has limitations that should be kept in mind when interpreting the results. The retrospective design introduces potential selection biases, and the relatively small number of confirmed posterolateral meniscocapsular detachments (*n* = 8) resulted in wide confidence intervals for sensitivity estimates, limiting the precision of this parameter. No dedicated PLC-oriented sequences were acquired, and standard slice thicknesses were employed throughout, potentially underestimating the true detection capability of MRI under optimized protocols. Variability in arthroscopic documentation of subtle posterolateral lesions represents an additional source of uncertainty, as the thoroughness of posterolateral exploration may differ between surgeons. In addition, surgeons were not blinded to the preoperative MRI findings, as review of MRI formed part of routine clinical practice. This may have introduced incorporation bias by increasing awareness of suspected posterolateral lesions during arthroscopy, potentially leading to an overestimation of MRI–arthroscopy concordance. Finally, the concordant-reader model adopted for the primary endpoint, while conservative and methodologically rigorous, may underestimate true MRI sensitivity when one reader correctly identifies a lesion that the other misses. Detailed clinical and temporal data were not systematically available for this retrospective cohort. As a partial proxy, the interval between MRI and arthroscopy could be calculated from institutional records for the 121 examinations initially screened: the mean interval was approximately 137 days (about 4.5 months), with a median of 89 days and a wide range of 6 to 775 days. This variability means that, for some patients, a substantial period elapsed between imaging and surgical correlation, during which meniscal or capsular findings could in principle have evolved; this should be considered when interpreting MRI–arthroscopy concordance. Beyond this interval, other clinically relevant variables—mechanism of injury, primary ACL injury versus graft failure as separate strata, presence of lateral-sided symptoms, and degree of clinical instability—were not consistently recorded and could not be analyzed; their potential influence on detachment detection remains unaddressed. Finally, although the inclusion of patients with ACL graft failure alongside native ACL ruptures was not expected to affect assessment of the posterolateral compartment itself, this postoperative subgroup may introduce heterogeneity related to prior meniscal treatment or, where present, adjacent fixation hardware, which we did not separately quantify.

Looking ahead, the most pressing need is a prospective study comparing standard protocols with dedicated posterolateral sequences—thin-slice oblique acquisitions or high-resolution isotropic 3D reformats—to quantify how much diagnostic gain is achievable with incremental protocol complexity. Multicentre collaboration would also allow more precise prevalence estimates, particularly given the low event rate that constrained our sensitivity analysis.

Taken together, our findings reinforce the idea that posterolateral meniscocapsular detachment is a demanding diagnosis on routine MRI—not because the findings are exotic, but because they are subtle and easily rationalised as artefact. Systematic posterolateral evaluation, familiarity with the anatomy of the three fascicles and their respective imaging behaviour, and a low threshold for suspicion in ACL-injured knees with unexplained lateral symptoms are, in our view, the most practical steps toward reducing underdiagnosis.

## 5. Conclusions

Posterolateral meniscocapsular detachment represents a subtle but clinically relevant component of the injury spectrum associated with ACL rupture. Routine MRI performed with standard protocols at 1.5 T and 3 T demonstrates good overall diagnostic performance for detecting these lesions when careful multiplanar assessment and detailed anatomical analysis are systematically applied. Knowledge of the complex and variable anatomy of the popliteomeniscal fascicles is essential, particularly regarding the frequent variability of the anteroinferior fascicle and the oblique orientation of the posteroinferior fascicle, which may simulate discontinuity due to partial volume averaging.

Radiologists should maintain a high degree of suspicion in ACL-injured knees presenting with lateral compartment symptoms, posterior horn abnormalities of the lateral meniscus, or imaging findings adjacent to the popliteal hiatus. Awareness of common diagnostic pitfalls, especially pseudotear appearances of the posteroinferior fascicle, is crucial to avoid both underdiagnosis and overcalling of pathology.

Accurate MRI characterization of posterolateral meniscocapsular lesions may have direct implications for arthroscopic management by guiding targeted surgical exploration and supporting careful intraoperative assessment of suspected posterolateral lesions. Whether improved recognition of these injuries translates into better restoration of rotational knee stability, fewer persistent postoperative symptoms, or a lower risk of ACL graft overload and long-term degenerative changes was not evaluated in this study and remains to be confirmed in prospective studies with postoperative clinical follow-up. Future prospective studies using dedicated thin-slice posterolateral MRI protocols are also warranted to further refine diagnostic criteria and optimize imaging assessment of the popliteomeniscal fascicular complex.

## Figures and Tables

**Table 1:** MRI protocol characteristics by magnetic field strength for the 117 examinations included in the diagnostic accuracy analysis.

Field Strength	*n* (%)	Sequences and Planes	Slice Thickness
1.5 T	97 (82.9%)	T1, T2, PD, STIR (sagittal, axial, coronal)	3–4 mm
3 T	20 (17.1%)	T1, T2, PD, STIR (sagittal, axial, coronal)	3–4 mm
Total	117 (100%)	No dedicated PLC-oriented sequences	—

Sequence composition was similar at both field strengths; the only systematic difference between groups was field strength itself, since both used comparable slice thickness and non-targeted imaging planes.

**Table 2:** Imaging features used to support a diagnosis of posterolateral meniscocapsular detachment and features favoring a pseudotear.

Features Favoring True Detachment	Features Favoring Pseudotear/Artifact
Frank fascicle discontinuity seen consistently across multiple imaging planes	Apparent discontinuity seen on only one plane, not confirmed on orthogonal images
Fluid signal within the meniscocapsular/popliteal hiatus space	Thin, oblique fascicle (especially the piPMF) cut tangentially by the imaging plane
Abnormal signal or distension within the popliteal hiatus	Preserved continuity on at least one orthogonal plane
Frank separation of the posterior horn of the lateral meniscus from the adjacent capsule	No associated fluid, edema, or meniscal displacement

These criteria are descriptive rather than formally validated and are intended to summarize the features considered by readers in this study.

**Table 3:** Diagnostic performance of MRI for posterolateral meniscocapsular detachment, using arthroscopy as the reference standard and a concordant-reader model (lesion considered present only when identified by both readers independently).

		Arthroscopic Findings		
		Lesion	No Lesion	Total
Readers’ assessment	Positive	7	10	17
	Negative	1	99	100
	Total	8	109	117

CI: confidence interval. Sensitivity: 7/8 = 87.5% (95% CI: 47.3–99.7%). Specificity: 99/109 = 90.8% (95% CI: 83.7–95.5%).

**Table 4:** Diagnostic performance of MRI for ramp lesions, using arthroscopy as the reference standard.

		Arthroscopic Findings		
		Ramp Lesion	No Ramp Lesion	Total
Readers’ assessment	Positive	22	11	33
	Negative	6	78	84
	Total	28	89	117

CI: confidence interval. Sensitivity: 22/28 = 78.6% (95% CI: 59.0–91.7%). Specificity: 78/89 = 87.6% (95% CI: 79.0–93.7%).

**Table 5:** Concordance between MRI and arthroscopy for the detection of concomitant meniscal tears (medial and/or lateral), categorized as complete concordance, partial concordance, or discordance.

Outcome	*n*/Total	Percentage	95% CI
Complete concordance	80/117	68.4%	59.1–76.7%
Partial concordance	16/117	13.7%	8.0–21.3%
Discordance	21/117	17.9%	11.5–26.1%

CI: confidence interval. Complete concordance: all arthroscopic findings correctly identified on MRI; partial concordance: at least one meniscal tear correctly identified but others missed or overcalled; discordance: no agreement between MRI and arthroscopic findings.

**Table 6:** Association between posterolateral meniscocapsular detachment and selected clinical and imaging variables. No statistically significant associations were identified.

Variable	No Detachment (*n* = 109)	Detachment (*n* = 8)	*p*-Value
Male sex	73 (67.0%)	6 (75.0%)	1.000
Age, mean ± SD (years)	30.7 ± 13.3	28.8 ± 17.5	0.433
Lateral tibial plateau edema	75 (68.8%)	6 (75.0%)	1.000

SD: standard deviation. *p*-values calculated using Fisher’s exact test (categorical variables) and independent-samples *t*-test (continuous variables). Statistical significance threshold: *p* < 0.05.

## Data Availability

All study data were retrospectively retrieved from the institutional database of Humanitas Research Hospital (Rozzano, Milan, Italy), including MRI examinations and corresponding intraoperative arthroscopic findings obtained from orthopedic surgical records.

## References

[1] Kurtoglu Z., Elvan Ö., Aktekin M., Çolak M. (2017). Morphological features of the popliteus tendon, popliteofibular and lateral (fibular) collateral ligaments. Int. J. Morphol..

[2] Kang K.T., Koh Y.G., Nam J.H., Jung M., Kim S.J., Kim S.H. (2019). Biomechanical evaluation of the influence of posterolateral corner structures on cruciate ligaments forces during simulated gait and squatting. PLoS ONE.

[3] Liu P., Gong X., Zhang J., Ao Y. (2020). Anatomic, all-arthroscopic reconstruction of posterolateral corner of the knee: A cadaveric biomechanical study. Arthroscopy.

[4] Peduto A., Nguyen A., Trudell D., Resnick D. (2008). Popliteomeniscal fascicles: Anatomic considerations using MR arthrography in cadavers. AJR Am. J. Roentgenol..

[5] Zappia M., Reginelli A., Chianca V., Carfora M., Di Pietto F., Iannella G., Mariani P.P., Di Salvatore M., Bartollino S., Maggialetti N. (2018). MRI of popliteo-meniscal fasciculi of the knee: A pictorial review. Acta Biomed..

[6] Aman Z.S., DePhillipo N.N., Storaci H.W., Moatshe G., Chahla J., Engebretsen L., LaPrade R.F. (2019). Quantitative and qualitative assessment of posterolateral meniscal anatomy: Defining the popliteal hiatus, popliteomeniscal fascicles, and the lateral meniscotibial ligament. Am. J. Sports Med..

[7] Zheng J., Xiao Q., Wu Q., Deng H., Zhai W., Lin D. (2021). Tears of the popliteomeniscal fascicles of the lateral meniscus: An arthroscopic classification. Cartilage.

[8] D’Addona A., Izzo A., Di Vico G., Rosa D., Maffulli N. (2021). The popliteomeniscal fascicles: From diagnosis to surgical repair: A systematic review of current literature. J. Orthop. Surg. Res..

[9] Dancy M.E., Tagliero A.J., Till S.E., Saris D.B., Levy B.A., Camp C.L., Krych A.J. (2023). Surgical repair of hypermobile lateral meniscus secondary to popliteomeniscal fascicle tears improves pain and mechanical symptoms. Arthrosc. Sports Med. Rehabil..

[10] Di Vico G., Simonetta R., Correra G., Corona K., Proietti L., Morris B.J., Cerciello S. (2023). Popliteomeniscal fascicles tears with lateral meniscus instability: Outcomes of arthroscopic surgical technique at mid-term follow-up. Arch. Orthop. Trauma Surg..

[11] Papalia R., Simonetta R., Di Vico G., Torre G., Saccone L., Espregueira-Mendes J., Denaro V. (2016). Tears of popliteomeniscal fascicles, diagnostic and clinical implications: A review of the evidence. Muscles Ligaments Tendons J..

[12] Mariani P.P., Torre G., Battaglia M.J., Ciatti R., Papalia R. (2024). Concomitant popliteomeniscal fascicles tears are found in 21% of professional soccer players with acute anterior cruciate ligament injuries. Arthrosc. Sports Med. Rehabil..

[13] Bhatia S., LaPrade C.M., Ellman M.B., LaPrade R.F. (2014). Meniscal root tears: Significance, diagnosis, and treatment. Am. J. Sports Med..

[14] Flury A., Hodel S., Andronic O., Kaiser D., Fritz B., Imhoff F.B., Fucentese S.F. (2023). Extent of posterolateral tibial plateau impaction fracture correlates with anterolateral complex injury and has an impact on functional outcome after ACL reconstruction. Knee Surg. Sports Traumatol. Arthrosc..

[15] Moteshakereh S.M., Zarei H., Nosratpour M., Moshfegh M.Z., Shirvani P., Mirahmadi A., Mahdavi M., Noshahr R.M., Farrokhi M., Kazemi S.M. (2024). Evaluating the diagnostic performance of MRI for identification of meniscal ramp lesions in ACL-deficient knees: A systematic review and meta-analysis. J. Bone Jt. Surg. Am..

[16] Cristiani R., van de Bunt F., Kvist J., Stålman A. (2023). High prevalence of meniscal ramp lesions in anterior cruciate ligament injuries. Knee Surg. Sports Traumatol. Arthrosc..

[17] Nonaka S., Hatayama K., Tokunaga S., Kakiage H., Hirasawa S., Terauchi M., Chikuda H. (2024). Diagnostic accuracy of MRI in the 120° flexed-knee position for detecting and classifying meniscal ramp lesion. Am. J. Sports Med..

[18] Beel W., Macchiarola L., Mouton C., Laver L., Seil R. (2022). The hypermobile and unstable lateral meniscus: A narrative review of the anatomy, biomechanics, diagnosis and treatment options. Ann. Jt..

[19] Keyhani S., Movahedinia M., Soleymanha M., Verdonk R., Kazemi M., Qoreishy M. (2021). Repair of popliteomeniscal fascicles tear using a posterior transseptal portal fixes hypermobile lateral meniscus. J. Exp. Orthop..

[20] Vosoughi F., Mafhoumi A., Gouravani M., LaPrade R.F., Vaziri A.S., Movahedinia M., Keyhani S. (2024). Hypermobile lateral meniscus: A systematic review of current treatment options. Knee Surg. Sports Traumatol. Arthrosc..

[21] Chahla J., Moatshe G., Dean C.S., LaPrade R.F. (2016). Posterolateral corner of the knee: Current concepts. Arch. Bone Jt. Surg..

[22] Razi M., Safar Cherati A., Dadgostar H., Ahadi K., Razi H., Razi S., Soleimani M. (2024). Arthroscopic-assisted posterolateral corner reconstruction of the knee: Novel technique, classification, surgical algorithm, and midterm results. Arch. Bone Jt. Surg..

[23] Rakhra K.S., Delorme J.P., Sanders B., Liew A. (2022). The diagnostic accuracy of MRI for evaluating the posterolateral corner in acute knee dislocation. Eur. Radiol..

[24] Rosas H. (2016). Unraveling the posterolateral corner of the knee. Radiographics.

